# Vascular Gap Junctions Contribute to Forepaw Stimulation-Induced Vasodilation Differentially in the Pial and Penetrating Arteries in Isoflurane-Anesthetized Rats

**DOI:** 10.3389/fnmol.2018.00446

**Published:** 2018-12-03

**Authors:** Nobuhiro Watanabe, Satoshi Sasaki, Kazuto Masamoto, Harumi Hotta

**Affiliations:** ^1^Department of Autonomic Neuroscience, Tokyo Metropolitan Institute of Gerontology, Tokyo, Japan; ^2^Graduate School of Informatics and Engineering, The University of Electro-Communications, Tokyo, Japan; ^3^Brain Science Inspired Life Support Research Center, The University of Electro-Communications, Tokyo, Japan

**Keywords:** somatosensory stimulation, vasodilation, cerebral blood flow, gap junction, two-photon microscopy, laser speckle flowmetry, carbenoxolone, rat

## Abstract

Somatosensory stimulation causes dilation of the pial and penetrating arteries and an increase in cerebral blood flow (CBF) in the representative region of the somatosensory cortex. As an underlying mechanism for such stimulation-induced increases in CBF, cerebral artery dilation has been thought to propagate in the vascular endothelium from the parenchyma to the brain surface. Vascular gap junctions may propagate vasodilation. However, the contribution of vascular gap junctions to cerebrovascular regulation induced by somatosensory stimulation is largely unknown. The aim of the present study was to investigate the contribution of vascular gap junctions to the regulation of the pial and penetrating arteries during neuronal activity attributed to somatosensory stimulation. Experiments were performed on male Wistar rats (age: 7–10 weeks) with artificial ventilation under isoflurane anesthesia. For somatosensory stimulation, the left forepaw was electrically stimulated (1.5 mA, 0.5 ms and 10 Hz, for 5 s). The artery in the forelimb area of the right somatosensory cortex was imaged through a cranial window using a two-photon microscope and the diameter was measured. Carbenoxolone (CBX) was intravenously (i.v.) administered, at a dose of 100 mg/kg, to block vascular gap junctions. The forepaw electrical stimulation increased the diameter of the pial and penetrating arteries by 7.0% and 5.0% of the pre-stimulus diameter, respectively, without changing the arterial pressure. After CBX administration, the change in pial artery diameter during forepaw stimulation was attenuated to 3.2%. However, changes in the penetrating artery were not significantly affected. CBF was measured using a laser speckle flowmeter, together with somatosensory-evoked potential (SEP) recorded in the somatosensory cortex. The extent of CBF increase (by 24.1% of the pre-stimulus level) and amplitude of SEP were not affected by CBX administration. The present results suggest that vascular gap junctions, possibly on the endothelium, contribute to pial artery dilation during neuronal activity induced by somatosensory stimulation.

## Introduction

Regional cerebral blood flow (CBF) in the sensory cortices increases when local neurons are activated by somatosensory stimulation. Such a CBF change is used as an indicator of neuronal activity in brain imaging techniques, such as functional magnetic resonance imaging (Ogawa et al., 1990, 1992; Logothetis, 2002; Hotta et al., 2014; Fukuda et al., 2016; Poplawsky et al., 2017); however, the mechanisms underlying CBF regulation have not been fully clarified. Therefore, to correctly understand the brain imaging data, it is important to clarify the mechanism of cerebrovascular regulation.

In the cerebral cortex, arterioles branching from the pial artery enter into the parenchyma (penetrating artery), further branch to the capillary, and supply blood to vital tissues. When CBF increases accompany neuronal activity in the primary somatosensory (SI) cortex, vasodilation occurs in the parenchymal artery and pial artery (Ngai et al., 1988; Ngai and Winn, 2002; Tian et al., 2010; Sekiguchi et al., 2014; Mishra et al., 2016). Ascending somatosensory information activates neurons in layer IV of the SI cortex and subsequent neural activation transmits to the neighboring layers, such as layer II/III (Helmstaedter et al., 2008; Guy and Staiger, 2017). Vasodilation induced by somatosensory stimulation is also thought to propagate from the parenchyma to the brain surface (Silva and Koretsky, 2002; Tian et al., 2010; Iadecola, 2017; Masamoto and Vazquez, 2018). A study showed that somatosensory stimulation-induced dilation of the pial artery occurs from the distal to the proximal side towards the heart (i.e., the opposite direction to blood flow); however, after the vascular endothelium of the artery is transversely damaged by a dye-light method, vasodilatation is not transmitted over the injured site (Chen et al., 2014). Thus, the vascular endothelium is possibly involved in the propagation of vasodilatation at the brain surface. However, how the vascular endothelium propagates cerebral vasodilatation information has not yet been clarified.

There are gap junctions between vascular endothelial cells, smooth muscles and endothelial and smooth muscle cells, and electrical signals spread from cell to cell via gap junctions (de Wit and Griffith, 2010). To date, a study using the hamster cheek pouch artery showed that propagation of vasodilatation induced by local application of acetylcholine was attenuated or abolished by putative gap junction blockers, such as a hypertonic sucrose solution and octanol (Segal and Duling, 1989). On the other hand, the involvement of cerebrovascular gap junctions in vascular regulation during somatosensory stimulation remains unknown. Therefore, the present study aimed to elucidate the involvement of gap junctions of the cerebral vasculature in cerebrovascular regulation during neuronal activity induced by somatosensory stimulation. For this purpose, we investigated the effect of intravenous (i.v.) administration of a gap junction blocker, carbenoxolone (CBX), at a dose that does not act on the cerebral parenchyma.

It has been suggested that mechanisms of cerebrovascular regulation differ between the brain surface and parenchyma (Adachi et al., 1992; Petzold and Murthy, 2011; Hotta et al., 2013; Hotta, 2016). Therefore, the contribution of gap junctions needs to be investigated for both the pial and penetrating arteries. An imaging technique with a two-photon microscope enables the kinetic observation of parenchymal blood vessels *in vivo* (Hotta et al., 2013; Sekiguchi et al., 2014; Ito et al., 2017). In the present study, the pial and penetrating arteries were imaged using a two-photon microscope and the change in artery diameter in response to somatosensory stimulation was measured. Furthermore, CBF was measured with a laser speckle flowmeter in different animal groups.

## Materials and Methods

### Animals

In the present study, experiments were conducted on 15 Wistar male rats (age: 7–10 weeks). Ten animals were used for cerebrovascular imaging using a two-photon microscope and five were used for CBF measurement using a laser speckle flowmeter. All experimental protocols were approved by the animal care and use committee of the Tokyo Metropolitan Institute of Gerontology (animal ethics committee: approval number 17025) and conformed to the “Guidelines for proper implementation of animal experiments” established by the Japan Society for the Promotion of Science in 2006.

Rats were anesthetized with isoflurane (Escain, Mylan Inc., Canonsburg, PA, USA). Isoflurane was vaporized by room air or mixed gas (30% O_2_, 70% N_2_). The inhalation concentration of isoflurane was adjusted to 4% for anesthesia induction and maintained at 2%–3.5% during surgery. During data recording, isoflurane was maintained at 1.5%–1.7%, which was sufficient to eliminate the corneal reflex. Catheters were implanted in the femoral artery to continuously record arterial pressure and in the femoral vein to administer drugs and supplemental fluids. The trachea was intubated and rats were artificially ventilated (SN-480-7; Shinano Seisakusyo, Tokyo, Japan). Respiration was adjusted to maintain end-tidal CO_2_ levels at 3.5%–4.0% (Microcap, Oridion Medical, Jerusalem, Israel). Rectal temperature was maintained at 37.0–37.5°C using a feedback-regulated temperature control system. The obtained blood pressure (BP) waveform was digitized at 1,000 Hz (Micro1401mkII; Cambridge Electronic Design, Cambridge, UK) and stored on a personal computer. Mean arterial pressure (MAP) was calculated with a time constant of 1 s (Spike 2 ver 8.03; Cambridge Electronic Design, Cambridge, UK).

### Somatosensory Stimulation

For somatosensory stimulation, electrical stimulation was applied to the left forepaw (SEN-8203 with SS-203J, Nihon Kohden, Tokyo, Japan). Two 30G needles were inserted in the 2nd/3rd toes and 3rd/4th toes to a depth of 5 mm, and current was applied between the two electrodes (1.5 mA, 0.5 ms of pulse width, 10 Hz for 5 s; Masamoto et al., 2007). The interval between the start of the stimulation periods was separated by 40 s.

### Cranial Window Preparation for Two-Photon Imaging

The animal’s head was fixed to a stereotaxic instrument with ear bars (SR-5R-S, Narishige, Tokyo, Japan). On the day of the experiment, a cranial window (4 × 4 mm) was made on the right somatosensory cortex. The position of the cranial window was at 2 mm rostral and caudal to Bregma and at 2–6 mm lateral to the midline. The skull was partly excised using a dental drill. The dura mater was kept intact and protected with 2% agarose (Type III-A, High EEO, Sigma-Aldrich Co., St. Louis, MO, USA), dissolved in saline. Dental cement was applied around the cranial window to retain sufficient saline for the immersion objective lens. To reinforce the bonding of the dental cement to the skull, a screw was mounted in the occipital bone.

### Optical Mapping

**Figure 1:**
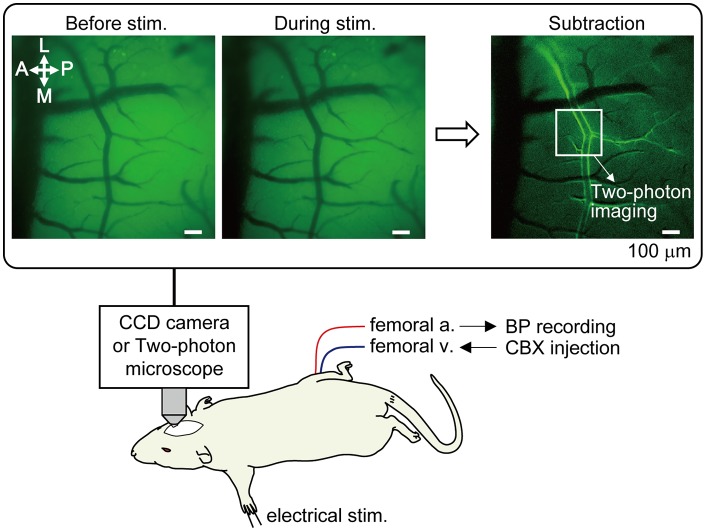
Method of optical mapping for identifying the somatosensory cortex region where arterial dilation is induced by forepaw stimulation. The right somatosensory cortex was imaged through a cranial window with a CCD camera. The left forepaw was electrically stimulated (1.5 mA, 0.5 ms of pulse duration, 10 Hz for 5 s). Four trials were performed and images from the four trials were averaged. Example images are shown in the rectangle; before and during forepaw stimulation (averaged over 5 s) and following subtraction. In the subtraction image, the bright border on the bilateral edges of the artery indicates the cortical region where arterial dilation was induced. The location indicated by a white square in the subtraction image was chosen for subsequent two-photon imaging. Note: a vein located on the anterior (rostral) side in the image and its branches do not dilate. Scale bar = 100 μm. BP, blood pressure; CBX, carbenoxolone.

Cerebral blood vessels were imaged using a fluorescence microscope (TCS SP 8 MP; Leica Microsystems GmbH, Wetzlar, Germany). Prior to two-photon imaging, optical mapping was first performed in order to narrow down the SI region where the arteries were most dilated in response to left forepaw stimulation (Figure 1). The cortex was illuminated with blue light (bandpass filter: 450–490 nm) and reflected light was captured with a CCD camera with a long pass filter (>515 nm). Using a 10× water immersion lens [numerical aperture (NA) = 0.30, Leica Microsystems], a region of 1.95 mm × 1.46 mm was imaged, and spatial resolution was 1.4 μm of the in-plane pixel size. An image was taken at a rate of 3.97 frames per second. Somatosensory stimulation was performed for four trials at 40 s intervals. An average image of four trials was created and a focused cortical area was determined by subtracting the images before and during forepaw stimulation (images enclosed with a rectangle frame in Figure 1).

### Cerebral Vasculature Imaging Using a Two-Photon Microscope

Rhodamine-labeled Ficoll (MW 70 kD, FC70-RB-1, Nanocs Inc., New York, NY, USA) dissolved in saline (5% solution) was administered via the femoral vein immediately before two-photon imaging. At the start of two-photon imaging, the penetrating arteries that dilated in response to forepaw stimulation were identified, based on the SI region determined by optical mapping. Furthermore, the upstream (pial) artery of the identified penetrating artery was also imaged. The penetrating artery was imaged at a depth of 60–80 μm from the brain surface. A three-dimensional image was taken to confirm that the penetrating arteries were diving in the parenchyma. The fluorescent dye administered was excited by a two-photon laser (850–880 nm; Chameleon Vision II, Coherent, Santa Clara, CA, USA) with a 25× correction collar lens (NA = 1.00, Leica Microsystems). The emission signal was detected on an external detector via a bandpass filter (585/40 nm). Sulforhodamine 101 (Sigma-Aldrich Co., St. Louis, MO, USA) dissolved with saline (0.3% solution) was intraperitoneally (i.p.) administered as needed to enhance the fluorescence signal. One plane image consisted of 512 × 512 pixels and the pixel size was 0.18–0.69 μm, depending on the digital zoom factor. An image was taken at a rate of 3.8–7.5 frames per second. Somatosensory stimulation was applied at 40-s intervals for eight trials. The imaging acquisition was synchronized with the electrical stimulator with the TTL signal in order to control the beginning of image acquisition.

### Analysis of Two-Photon Imaging Data

The obtained images were analyzed offline using custom-written MATLAB code (The Math Works Inc., Natick, MA, USA; Sekiguchi et al., 2013). Briefly, a rectangular region of interest was placed on the captured two-photon image, a medial filter was applied with 3 × 3 pixels, and the image was binarized by adjusting the threshold intensity manually. For the pial artery, the vessel diameter was obtained by dividing the area of the parallelogram conforming to the shape of the blood vessel by the length of the long axis of the parallelogram (that is, the axial direction of the blood vessel). For the penetrating artery, the diameter of the blood vessel was obtained by measuring the cross-sectional diameter of the blood vessel. Extracted values of vessel diameter were temporally smoothed with a time constant of 1 s and resampled at 2 Hz (Spike 2).

### Cerebral Blood Flow Measurement Using a Laser Speckle Flowmeter

Similar to the two-photon imaging experiment, the rat’s head position was fixed to a stereotaxic instrument. A cranial window was made by thinning the skull over the right somatosensory cortex using a dental drill until the underlying blood vessels were visible. To prevent drying, liquid paraffin oil was applied to the thinned bone. CBF was measured using a laser speckle flowmeter (moor LFPI; Moor Instruments, Devon, UK), consisting of an infrared semiconductor laser (wavelength at 785 nm) and a CCD camera (Hotta et al., 2011; Uchida and Kagitani, 2018). The laser speckle flowmeter measures parenchymal flow transcranially through the intact dura (Briers and Webster, 1996; Shih et al., 2012; Davis et al., 2014). The device was placed above the dorsal head of the rat, and the zoom was adjusted to include the forelimb region of the SI cortex and a field of view was approximately 66.5 mm^2^ (9.5 mm × 7 mm). One plane image consisted of 152 × 113 pixels, and the pixel size was about 62 μm. Images were acquired at a rate of 25 frames per second with 4 ms of exposure time.

### Analysis of Blood Flow Data

In order to quantify the temporal change in the regional CBF, blood flow data were extracted by placing a region of interest with a diameter of 1 mm on the right SI region where the blood flow changed most (moorFLPI Review V5.0, Moor Instruments). The extracted CBF data were temporally smoothed with a time constant of 1 s and resampled at 2 Hz (Spike 2).

### Recording of Somatosensory Evoked Potential

In experiments measuring CBF using a laser speckle flowmeter, the somatosensory-evoked potential (SEP) induced by forepaw stimulation was also recorded. A tungsten electrode (impedance 1 MΩ) was placed on the edge of the cranial window (UJ-70-0.2-1, Unique Medical, Tokyo, Japan). As a reference, a screw was mounted in the occipital bone. The electrical signal was amplified 1,000 times (MEG-6100, Nihon Kohden, Tokyo, Japan) and filtered (bandpass filter: 1.5–100 Hz). The amplified signal was digitized at 2,000 Hz (Micro1401mkII) and stored on a personal computer for offline analysis. The SEP elicited by the electrical stimulation was averaged 400 times and the amplitude of P1 and N1 was measured (Staba et al., 2003; Masamoto et al., 2009; Baker et al., 2013).

### Drugs

In order to investigate whether vascular gap junctions are involved in vasodilatation and CBF increases induced by somatosensory stimulation, CBX disodium salt (Sigma-Aldrich Co., St. Louis, MO, USA) solution (100 mg/mL in saline) was administered i.v. at a dose of 100 mg/kg. This administration dose and route was determined based on previous studies showing that CBX was not detected from cerebrospinal fluid following systemic administration (at 50 mg/kg; i.p.; Leshchenko et al., 2006) whereas vascular endothelium-dependent vasodilation was suppressed (Lan et al., 2011).

### Statistical Analysis

The Kolmogorov-Smirnov test showed that the data were not normally distributed and so we used non-parametric tests for our statistical analyses. The time course of blood vessel diameter, CBF and MAP were analyzed by a Friedman’s test followed by Dunn’s multiple comparisons test. The effects of CBX administration on vessel diameter, CBF, MAP and SEP were tested using a Wilcoxon signed-rank matched-paired test. Statistical analysis software was used (Prism 6; GraphPad Software Inc., La Jolla, CA, USA). For all statistical analyses, differences with a *p* < 0.05 were deemed statistically significant. Data are expressed as the median and interquartile range (25%–75%).

## Results

### Arterial Dilation Induced by Forepaw Stimulation

**Figure 2:**
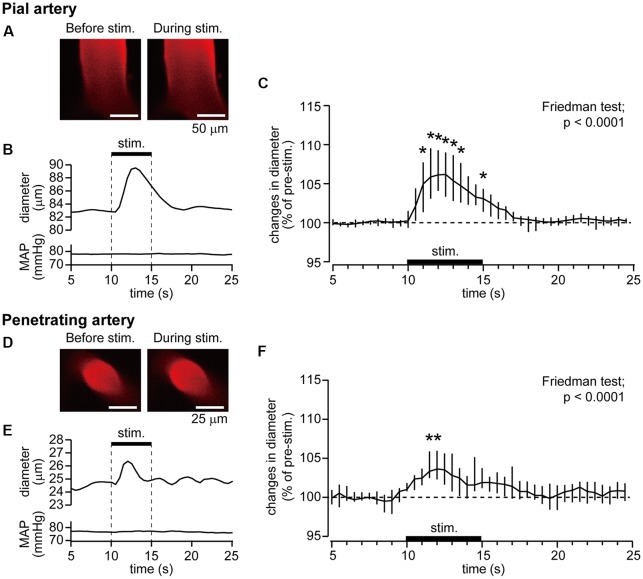
Forepaw stimulation causes the dilation of the pial and penetrating arteries without mean arterial pressure (MAP) changes. Blood vessels in the right somatosensory cortex were imaged through a cranial window with a two-photon microscope. Example images of the pial artery **(A)** and time course of changes in artery diameter and MAP **(B)** obtained from an individual rat are shown. Group data of diameter changes in the pial artery (*n* = 10) are presented **(C)**. Example images of the penetrating artery **(D)**, time course of changes in artery diameter and MAP **(E)** of a rat are shown. Group data of diameter changes in the penetrating artery (*n* = 8) are presented **(F)**. The thick horizontal bar indicates the period of forepaw stimulation (1.5 mA, 0.5 ms, 10 Hz for 5 s). Group data in **(C,F)** are expressed as a percentage of the pre-stimulation values. Asterisks (*) indicate a significant difference from a value at 9.5 s (*p* < 0.05). Data are expressed as median (interquartile range). Note: example data of the pial and penetrating arteries were obtained from the same animal. Scale bars = 50 μm and 25 μm in **(A,D)** respectively.

Example data of diameter changes in the pial artery (Figures 2A,B) and penetrating artery (Figures 2D,E) and MAP in response to forepaw stimulation are presented. The pial artery started to dilate at 1 s after the onset of the forepaw stimulation and peaked at 3 s. Dilation of the artery was then attenuated and returned to the pre-stimulation level by about 5 s after the end of stimulation. There was no apparent change in MAP during stimulation. The penetrating artery exhibited a similar change, began to dilate at 1 s after the onset of stimulation, peaked at 2 s, and gradually returned to the pre-stimulation level. When two-photon imaging was repeated within 40 min, with forepaw stimulation applied at 40-s intervals for eight trials (*n* = 3 for each of the pial and penetrating arteries), arterial diameter changes induced by the stimulation were not attenuated over time.

The pre-stimulation diameter was 57.5 μm (25.2–75.5 μm) for the pial artery (*n* = 10) and 25.4 μm (17.8–27.7 μm) for the penetrating artery (*n* = 8). Figures 2C,F show group data of changes in the diameters of the pial and penetrating arteries in response to forepaw stimulation. Time course data were plotted every 0.5 s. The Friedman test showed a statistically significant change for both the pial and penetrating arteries (*p* < 0.0001), whereas the forepaw stimulation did not affect MAP.

### Effect of CBX on Arterial Diameter Changes Induced by Forepaw Stimulation

**Figure 3:**
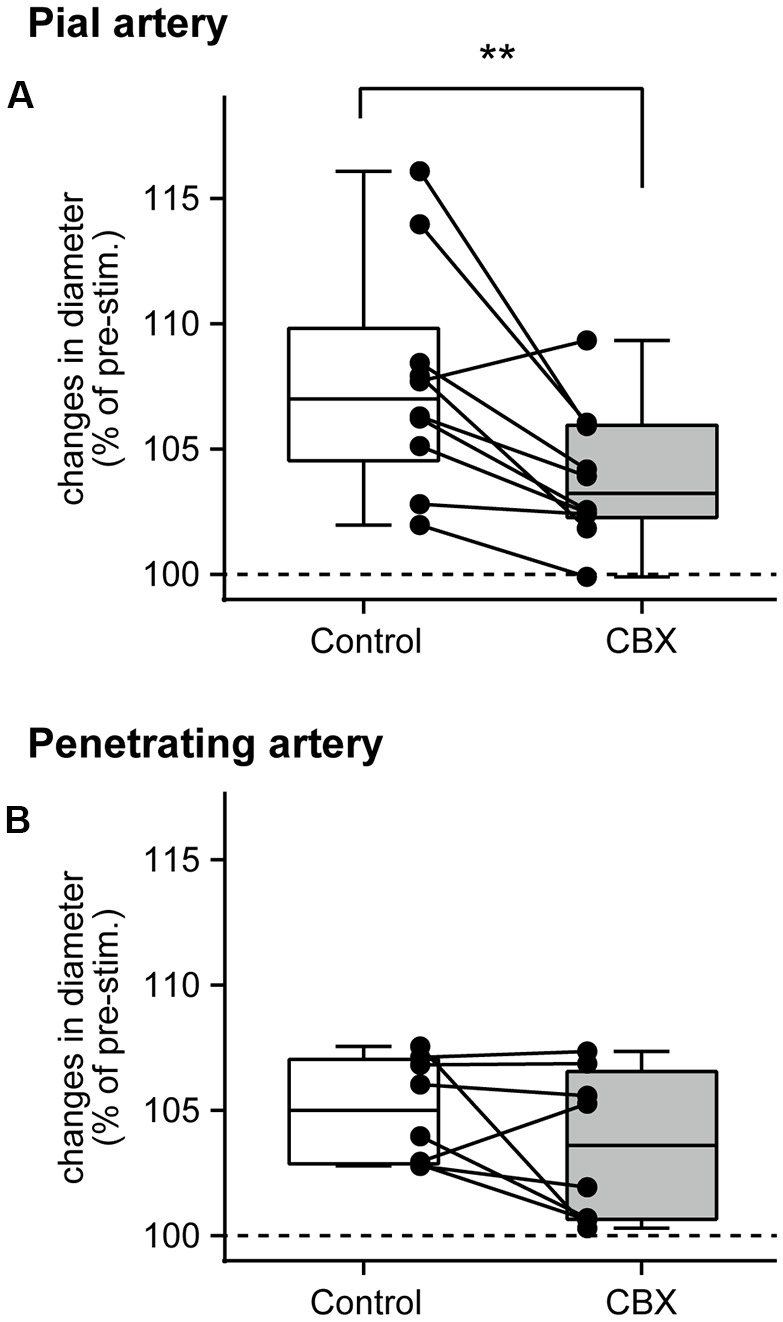
Gap junction blocker, CBX, administration attenuates pial artery dilation, but not penetrating artery dilation. Maximal changes in the diameter of the pial **(A)** and penetrating **(B)** arteries were compared before (Control) and after CBX administration. Values are expressed as a percentage of the pre-stimulation values. Individual closed circles and lines indicate data obtained from each rat. A box indicates the median and interquartile range, and whiskers indicate minimum and maximum values. Asterisks (**) indicate a significant difference (*p* < 0.01).

The peak arterial diameter change during forepaw stimulation was compared before and after CBX administration. Comparisons in each rat revealed that CBX administration attenuated the stimulation-induced increases in pial artery diameter in most rats (Figure 3A). The Wilcoxon matched-pairs signed rank test revealed that the stimulation-induced increase in pial artery diameter (107.0%, 104.6%–109.8%) was significantly attenuated after CBX administration (*p* = 0.0059; 103.2%, 102.3%–106.0%). In contrast, there was no consistent effect of CBX on increases in the penetrating artery diameter across individual rats (Figure 3B). The Wilcoxon matched-pairs signed rank test showed that there was no significant difference before or after CBX administration (*p* = 0.25, before CBX administration: 105.0%, 102.9%–107.0%; after CBX administration: 103.6%, 100.7%–106.6%).

The pre-stimulation arterial diameter was compared before and after CBX administration. The pial artery diameter was slightly but statistically significantly increased after CBX administration (*p* = 0.0059; before CBX administration: 57.5 μm, 25.2–75.5 μm; after CBX administration: 57.6 μm, 27.5–83.0 μm). On the other hand, no significant difference was observed in the penetrating artery (*p* = 0.62; before administration of CBX: 25.4 μm, 17.8–27.7 μm; after CBX administration: 24.9 μm, 17.7–31.6 μm).

### Effect of CBX Administration on Forepaw Stimulation-Induced Cerebral Blood Flow Increases

**Figure 4:**
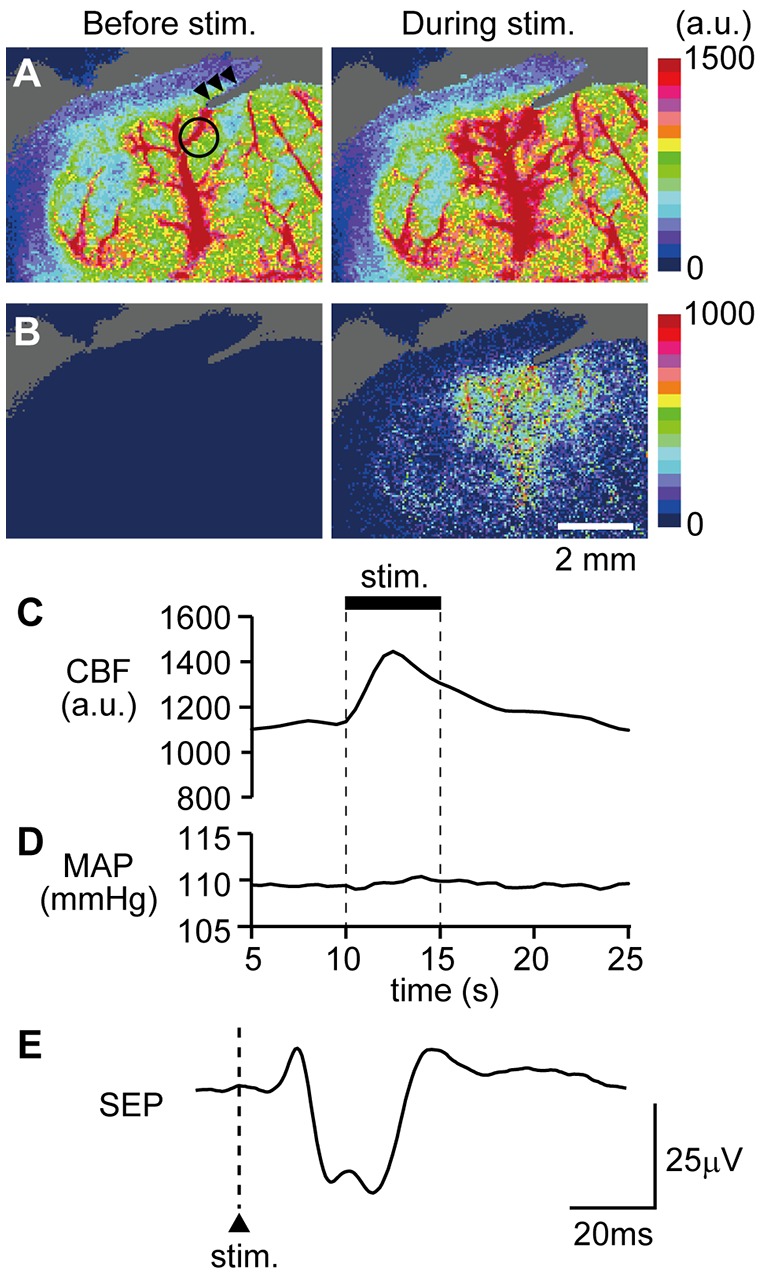
Example data showing that forepaw stimulation increases cerebral blood flow (CBF) and neural activity. CBF in the right somatosensory cortex was imaged through a cranial window with a laser speckle flowmeter. Example images before and during forepaw stimulation **(A)** and subtraction images **(B)** are presented. A circle in the left image of **(A)** indicates the location of a region of interest (1 mm in diameter) to extract CBF values and arrow heads indicate the artifact from a recording electrode for the somatosensory-evoked potential (SEP). Time courses of changes in CBF **(C)** and MAP **(D)** and averaged SEP **(E)** are shown. For **(C,D)**, the stimulation period is expressed as a thick horizontal bar. A triangle and vertical dash line in **(E)** indicate the onset of electrical stimulation applied to the forepaw. a.u.; arbitrary unit. Scale bars = 2 mm.

**Figure 5:**
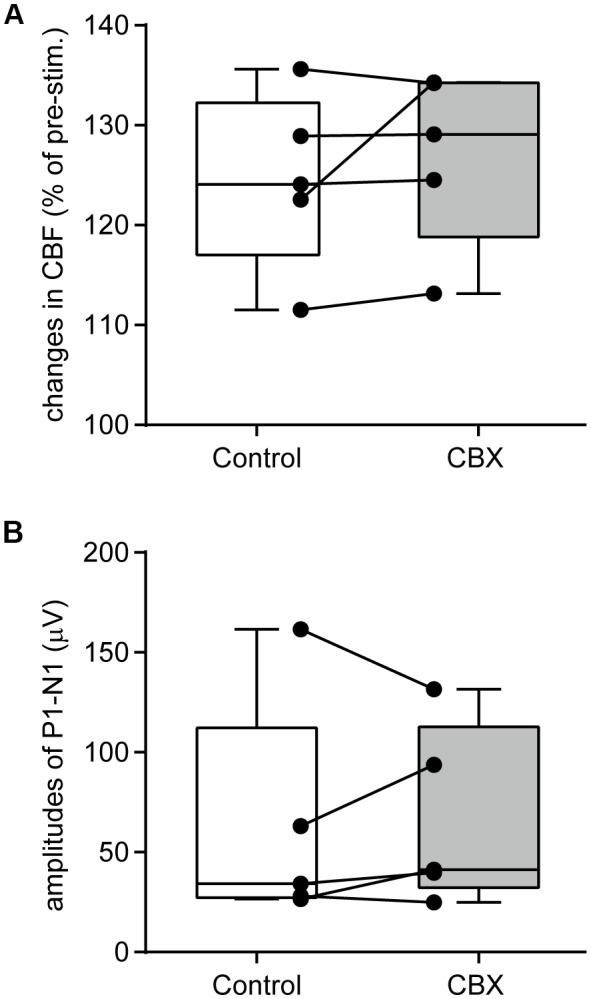
CBX administration does not affect CBF changes or neural activity. A maximal change in CBF **(A)** and the amplitudes of SEP (P1–N1; **B**) are compared before (Control) and after CBX administration. CBF values are expressed as a percentage of the pre-stimulation values. Individual closed circles and lines indicate data obtained from each rat. A box indicates the median and interquartile range, and whiskers indicate minimum and maximum values.

An example image of CBF measurement on the dorsal aspect of the right cerebral cortex is shown in Figure 4A. Following stimulation of the left forepaw, CBF locally increased in the right SI region (Figure 4B). Blood flow data were extracted by placing a region of interest over the SI forelimb area, showing that CBF increased to its maximum at 2.5 s after the onset of stimulation and gradually returned to the pre-stimulation level (Figure 4C). In contrast, MAP did not change (Figure 4D). The electroencephalogram was recorded on the edge of the cranial window and the SEP was elicited by forepaw stimulation (Figure 4E). The maximal increase in CBF and the amplitude of SEP during forepaw stimulation were compared before and after CBX administration (Figures 5A,B, respectively). The Wilcoxon matched-pairs signed rank test revealed that the CBF increase (124.1%, 117.0%–132.3%) did not differ after CBX administration (*p* = 0.31; 129.1%, 118.8%–134.3%). Similarly, the SEP amplitude was not different following CBX administration (*p* = 0.63; before CBX administration: 34.3 μV, 27.3–112.3 μV; after CBX administration: 41.3 μV, 32.3–112.7 μV).

The pre-stimulation CBF values (arbitrary unit, a.u.) were compared before and after CBX administration. There was no difference in the CBF values between the conditions (*p* = 0.81; before CBX administration: 1168 a.u., 1124–1320 a.u.; after CBX administration: 1182 a.u., 1146–1255 a.u.).

### Influence of CBX Administration on Resting Arterial Pressure

To test the influence of CBX on systemic arterial pressure, pre-stimulation MAP was compared before and after CBX administration. No significant difference in MAP was revealed by the Wilcoxon matched-pairs signed rank test (*p* = 0.31, before CBX administration: 78.9 mmHg, 73.7–88.5 mmHg; after CBX administration: 81.8 mmHg, 76.4–101.3 mmHg), showing that CBX administration in the present study did not affect systemic arterial pressure.

## Discussion

The present study shows that electrical stimulation of the forepaw dilated the pial and penetrating arteries and increased the regional CBF in the SI forelimb area contralateral to the stimulation, consistent with previous studies (Ngai et al., 1988; Ngai and Winn, 2002; Durduran et al., 2004; Dunn et al., 2005; Royl et al., 2006; Piché et al., 2010; Sekiguchi et al., 2014; Mishra et al., 2016). Further, the present study confirmed that arterial dilation and hyperemia were induced by forepaw stimulation at an intensity that did not affect arterial pressure. Therefore, the vasodilation and hyperemia induced by somatosensory stimulation in the present study were not due to systemic BP changes.

The novel finding of this study was that the dilative change in the pial artery was attenuated by CBX administration (100 mg/kg; i.v.), which was used to block the gap junctions of blood vessels. The dose and route of CBX administration was determined based on previous studies (Leshchenko et al., 2006; Lan et al., 2011). One study showed that CBX was not detected in the cerebrospinal fluid following systemic administration at a similar dose (50 mg/kg; i.p.; Leshchenko et al., 2006), indicating that CBX is unlikely to permeate the blood-brain barrier. On the contrary, there are several reports that systemic CBX administration suppresses epileptic seizure (100 mg/kg i.p.; Hosseinzadeh and Nassiri Asl, 2003; 20 mg/kg i.v.; Gareri et al., 2004) and enzymatic activity in the brain (100 mg/kg i.p.; Jellinck et al., 1993). These studies suggest that CBX penetrates the blood-brain barrier. Such a contradiction may be explained by a difference in experimental condition: anesthetized (Leshchenko et al., 2006; the present study) vs. awake (Jellinck et al., 1993; Hosseinzadeh and Nassiri Asl, 2003; Gareri et al., 2004) conditions. A study demonstrated that isoflurane decreases blood-brain barrier permeability by approximately 50% (Chi et al., 1992). Thus, CBX unlikely penetrates the blood-brain barrier under the present experimental condition. Additionally, it has been reported that arterial pressure was increased by intracerebroventricular administration of CBX (Tamura et al., 2011) and synaptic transmission was influenced by CBX application *in vitro* (Tovar et al., 2009). However, in the present study, CBX did not affect arterial pressure or the SEP amplitude. Hence, our results may suggest that administered CBX did not act on the brain parenchyma, but possibly on the cerebral vasculature. It has been reported that vasodilation induced by ischemia-reperfusion (due to nitric oxide generated from vascular endothelial cells) in the brachial artery is attenuated by systemic administration of CBX (Lan et al., 2011), indicating that systemically administered CBX acts on the vascular endothelium. Therefore, the present study results may support the hypothesis that pial arterial dilation associated with neuronal activity propagates through gap junctions of the cerebral vascular endothelium.

Limitations of the present study are that gap junctions are present in different types of cells including vascular endothelium and smooth muscles as well as glia (Sáez et al., 2005; Decrock et al., 2015; Manjarrez-Marmolejo and Franco-Pérez, 2016; Mayorquin et al., 2018) and that CBX affects not only gap junctions but also connexin hemichannels and pannexins, and neurotransmitter release mediated by volume regulated anion channels and P2X_7_ receptors (Rouach et al., 2003; Spray et al., 2006; Suadicani et al., 2006; Ye et al., 2009; Manjarrez-Marmolejo and Franco-Pérez, 2016). Studies using two-photon imaging report that topical application of CBX to the brain (100 μM) virtually stopped spreading fluorescent dye in putative glial cells (Nimmerjahn et al., 2004) and almost abolished glial cell activity during epileptic seizure (Baird-Daniel et al., 2017). However, it was shown that Ca^2+^ rise in astrocytes is slower than neural activity-induced hyperemia and vasodilation (Petzold and Murthy, 2011; Baird-Daniel et al., 2017; Gu et al., 2018). Thus, astrocytes may not contribute to fast responses like the neuronal activity-induced cerebrovascular dilation that the present study showed. In contrast, perfusion on the brain surface of selective blockers against connexins 43 and 37, representing the gap junctions expressed on astrocytes, attenuated the dilation of the pial artery induced by sciatic nerve stimulation (by approximately 75%) and epileptic seizures (by approximately 50%; Xu et al., 2008). We cannot exclude the possibility that the present dose (100 mg/kg) and route (i.v.) of CBX administration may also affect gap junctions in astrocytes. However, this is unlikely because the penetrating artery, which is surrounded by astrocytes, and parenchymal blood flow changes were not affected by CBX. Due to a wide spectrum effect of CBX, the present study is limited by the fact that the types of vascular cells contributing to the propagation of vasodilation cannot be specified. Therefore, further studies are needed to identify the types of cells involved in cerebrovascular regulation during neuronal activity.

In contrast to the pial artery, dilative changes in the penetrating artery during somatosensory stimulation were not affected by CBX. Differences in regulatory mechanisms between the pial and penetrating arteries were previously reported (Adachi et al., 1992; Petzold and Murthy, 2011; Hotta et al., 2013). For example, we have reported that electrical stimulation of the nucleus basalis of Meynert, where cholinergic neurons originate, dilates the penetrating artery, with little changes detected in the pial artery (Hotta et al., 2013). In cortical slice preparations, it has been reported that stimulation of single local cortical neurons affects the diameter of the nearby parenchymal artery (Cauli et al., 2004). We can assume that multiple mechanisms are simultaneously at play in the brain parenchymal arteries. The present results suggest that vascular gap junctions may have a role in the propagation of arterial dilation at the cortical surface level, though other mechanisms appear to predominate at a parenchymal level.

Although arterial dilation induced by somatosensory stimulation was attenuated by CBX administration in the pial artery, penetrating artery dilation and CBF increases were not affected. This is consistent with reports showing that changes in pial artery diameter do not reflect CBF changes, and that the penetrating artery is the major blood vessel determining parenchymal blood flow in the brain (Sekiguchi et al., 2014; Shih et al., 2015; Unekawa et al., 2017). Thus, what is the physiological significance of pial artery regulation via gap junctions? We assume that the dilation of the pial artery associated with neuronal activation may be helpful in reducing shear stress on the pial artery. When the penetrating artery dilates and blood flow velocity increases in the pial artery due to the suction effect, such blood flow velocity increases may be buffered by dilating the pial artery, explained by Bernoulli’s theory. It has been reported that arteriosclerosis prevails around bifurcation of blood vessels (Cheng et al., 2006; Baratchi et al., 2017). Therefore, dilation of the pial artery may be beneficial in protecting the pial artery itself by reducing shear stress. Another significant role of larger diameter arteries including the pial artery is to control blood perfusion into brain parenchyma in the case of a BP change (“autoregulation”). The cervical sympathetic nerve innervates the pial artery. Electrical stimulation of the nerve constricted the pial artery (Busija et al., 1982; Tamaki and Heistad, 1986) and reduced CBF increase during arterial pressure increases (Busija et al., 1980; Tamaki and Heistad, 1986; Waldemar et al., 1989). An *in vitro* study showed that the diameter of isolated middle cerebral artery is increased by gap junction blockers such as heptanol and 18α-glycyrrhetinic acid (Lagaud et al., 2002). Thus, gap junctions of the pial artery may also contribute to autoregulation of CBF.

The present results may implicate cerebrovascular dysfunction in some clinical conditions. For example, somatosensory stimulation-induced dilation of the pial artery is attenuated in streptozotocin-induced diabetic rats (Vetri et al., 2017). Additionally, cerebrovascular responses to visual stimulation and mental tasks are reduced in patients with type-II diabetes mellitus (Duarte et al., 2015; Nealon et al., 2017). A plausible explanation for such attenuations may be partly due to an impairment of gap junction function, since high glucose solution reduces expression and function of vascular gap junctions (Sato et al., 2002). However, some caution is necessary for applying the present findings to conscious humans, since the present study was conducted under anesthesia. Anesthesia use is advantageous: (1) to eliminate emotional factors, which may be affected by somatosensory stimulation (Sato et al., 1997), leading to cardiovascular function changes; and (2) to minimize movement artifact, which is critical for imaging experiments. Additionally, due to lowered blood-brain barrier permeability under anesthesia (Chi et al., 1992), the action site of the administered drug may be more limited. In contrast to these advantages, anesthesia may alter physiology related to cerebrovascular responses to neural activity; such as neural transmission, brain energy metabolism, vascular tone and glial functions (Conzen et al., 1992; Iida et al., 1998; Masamoto and Kanno, 2012). Hence, it is important to weight the advantages of performing experiments under anesthetized and non-anesthetized conditions.

## Conclusion

The present findings suggest that vascular gap junctions contribute to vasodilation of the pial artery but not focal responses of the parenchymal arteries during neuronal activation by somatosensory stimulation. Such vasodilation might be attributable to gap junctions on the vascular endothelium.

## Data Availability

The raw data supporting the conclusions of this manuscript will be made available by the authors, without undue reservation, to any qualified researcher.

## Author Contributions

NW, SS, KM and HH designed the research and prepared the manuscript. NW and SS performed experiments and analyzed data. All authors approved the final version of the manuscript and agreed to be accountable for all aspects of the work in ensuring that questions related to the accuracy or integrity of any part of the work are appropriately investigated and resolved.

## Conflict of Interest Statement

The authors declare that the research was conducted in the absence of any commercial or financial relationships that could be construed as a potential conflict of interest.

## Abbreviations

CBF: cerebral blood flow
CBX: carbenoxolone
i.p.: intraperitoneally
i.v.: intravenously
MAP: mean arterial pressure
NA: numerical aperture
SEP: somatosensory-evoked potential
SI: primary somatosensory.
